# Adjustment of Wettability in Biomaterials: Evaluation of the Impact of Red Propolis Fractions on Electrospun Poly(ε‐caprolactone)

**DOI:** 10.1002/cbdv.202501617

**Published:** 2025-11-29

**Authors:** Leonardo Sobreira Rodrigues, Emanuelly Carolyne Marques de Farias Nanes, Adriana Carla de Oliveira Lopes, Ticiano Gomes do Nascimento, Johnnatan Duarte de Freitas, Camila Braga Dornelas, Adriana Santos Ribeiro, Ligia Maria Manzine Costa

**Affiliations:** ^1^ Academic Unit Technology Center Federal University of Alagoas Maceió Alagoas Brazil; ^2^ Institute of Pharmaceutical Sciences University of Alagoas Maceió Alagoas Brazil; ^3^ Federal Institute of Alagoas Maceió Alagoas Brazil; ^4^ Department of Physics and Chemistry São Paulo State University of Júlio de Mesquita Filho Ilha Solteira São Paulo Brazil

**Keywords:** electrospinning, polycaprolactone, red propolis

## Abstract

This study demonstrates the modulation of wettability in biomaterials through the incorporation of distinct fractions of red propolis into electrospun poly(ε‐caprolactone) (PCL). Chromatographic analysis revealed that the hydroalcoholic extract contains a significantly higher concentration of active compounds, particularly flavonoids, compared to the wax fraction. The electrospinning process yielded fibrous mats with average fiber diameters below 900 nm, maintaining uniform morphology regardless of the type or concentration of the additive, as confirmed by scanning electron microscopy (SEM). Wettability assessments, performed via contact angle measurements and swelling tests, indicated that extract‐containing samples exhibited increased hydrophilicity, with contact angles below 90° at concentrations of 10% or higher, while wax‐containing formulations retained hydrophobic behavior. Cytotoxicity assays confirmed high cell viability across all formulations, indicating the absence of cytotoxic effects. Collectively, these findings demonstrate that the incorporation of different red propolis fractions modulates the structural and surface properties of electrospun PCL, supporting their potential for use in the development of customizable biomaterials for applications in tissue engineering and bioactive wound dressings.

## Graphical abstract

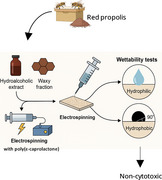

## 1 Introduction

Recent advances in biomaterials development have fostered the design of systems with specific properties tailored for clinical applications, such as bioactive wound dressings and scaffolds for tissue engineering [1, 2]. In this context, the electrospinning technique has gained prominence due to its ability to generate fibrous mats with high porosity and large surface area—key features for promoting cell–matrix interactions and facilitating the efficient diffusion of nutrients and therapeutic agents [3, 4, 5, 6].

Poly(ε‐caprolactone) (PCL) is extensively used in the fabrication of biomaterials owing to its biocompatibility, biodegradability, and favorable mechanical properties, making it a promising candidate for medical applications [7, 8, 9, 10]. Moreover, the modulation of surface characteristics, particularly wettability, is critical for tailoring interactions with the biological environment. Surfaces exhibiting contact angles below 90° are classified as hydrophilic and are beneficial for rapid exudate absorption, the maintenance of a moist environment conducive to healing, and the controlled release of therapeutic compounds. Conversely, surfaces with contact angles above 90° exhibit hydrophobic behavior and are suitable for applications requiring impermeable barriers, restricted drug diffusion, and the prevention of undesired adhesions [11, 12, 13].

Numerous studies have highlighted the potential of both hydrophobic and hydrophilic electrospun mats for targeted healthcare applications. Hydrophobic mats have demonstrated efficacy as protective barriers by preventing fluid and pathogen infiltration and enabling prolonged drug release through hydrophobic interactions. In contrast, hydrophilic mats have been widely explored in the development of wound dressings, as their high exudate absorption capacity and moisture retention significantly contribute to tissue regeneration and wound healing processes [11, 13, 14, 15, 16, 17, 18].

Furthermore, the literature underscores the biological significance of red propolis, particularly from the Alagoas region of Brazil, due to its remarkable antibacterial, antioxidant, wound healing, antifungal, anticancer, and anti‐inflammatory properties [19, 20, 21, 22, 23]. These bioactivities render red propolis an excellent additive for incorporation into biomaterial systems, with the potential to enhance biological performance and create synergistic interactions with polymeric matrices [24, 25, 26, 27].

This study proposes an innovative strategy involving the integration of Alagoas red propolis into electrospun PCL mats. In addition, it investigates the comparative effects of different propolis fractions and extracts on the modulation of surface properties, with a particular focus on wettability, aiming to develop customizable biomaterials. This approach seeks to optimize material–host interactions to address specific requirements in tissue engineering and bioactive wound care applications.

## 2 Experimental

This section outlines the materials and experimental procedures employed to develop electrospun PCL mats incorporated with different fractions of red propolis. The methodology includes the extraction and separation of hydroalcoholic and wax fractions from crude red propolis, preparation of polymeric solutions, and fabrication of fibrous mats via electrospinning. The resulting materials were subjected to morphological characterization by scanning electron microscopy (SEM), surface wettability assessments through contact angle and swelling tests, and cytotoxicity evaluation to determine cell viability.

### 2.1 Materials

PCL (Mw ∼80 000 g/mol, Sigma‐Aldrich, St. Louis, MO, USA), red propolis was collected from colonies of *Apis mellifera* L. located in beekeeping areas of Alagoas State, Brazil, chloroform (Synth, Diadema, Brazil), methanol (Synth, Diadema, Brazil), and ethanol (Neon, Suzano, Brazil) were used. All reagents were of analytical grade.

### 2.2 Preparation of Red Propolis Fractions

#### 2.2.1 Extraction of Red Propolis and Wax Fraction

**FIGURE 1:**
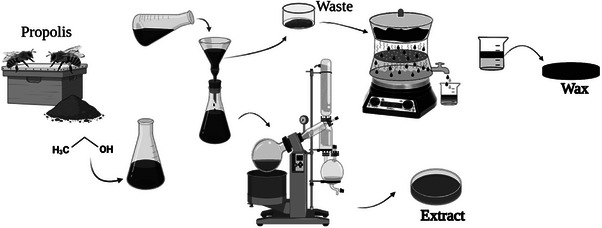
Schematic representation of the preparation of red propolis hydroalcoholic extract and wax fraction.

Raw red propolis was sourced from an apiary located in Paripueira (Maceió, Brazil), with geographic coordinates: 9°26.448′ S, 35°31.710′ W, and an elevation of 4.7 m above sea level. To obtain the concentrated hydroalcoholic extract, 100 g of crude red propolis were suspended in 400 mL of 70% ethanol (v/v), as illustrated in Figure 1. The suspension was left undisturbed at room temperature (27°C) for 48 h, followed by filtration through standard filter paper. The supernatant (hydroalcoholic extract) was collected and concentrated using a rotary evaporator (IKA RV10, Germany) at 50°C. The resulting extract was stored at 10°C for further use [20, 24].

The solid residue (waste) remaining after hydroalcoholic extraction comprised an insoluble resinous mass. This residue was oven‐dried at 50°C for 24 h to remove residual solvents. To extract the wax fraction, the dried material was subjected to decoction using hot deionized water at 100°C. This process was repeated until the aqueous phase appeared translucent. The wax‐rich material was then cooled and stored at room temperature (Figure 1).

#### 2.2.2 Preparation of PCL Mats Containing Red Propolis Extract and Wax

Samples were prepared by incorporating red propolis extract or wax at concentrations of 1%, 5%, 10%, and 15% (w/w) relative to the total weight (1.8 g) of PCL in the polymer solution [28]. A reference solution, labeled PCL, was prepared using only PCL without any additives. The other formulations were prepared by adding either extract or wax in the specified concentrations.

The selected concentration range (1%–15%) was determined based on preliminary electrospinning tests, which showed homogeneous dispersion and stable jet formation within this interval. Concentrations above 15% did not produce continuous fibers, leading to bead formation.

All components were dissolved in a standardized solvent system consisting of chloroform and methanol (20 mL, 4:1 v/v), as previously described by Costa et al. [28]. The mixtures were stirred at 27°C for 5 h to ensure complete homogenization.

After complete dissolution, each solution was electrospun using a high‐voltage power supply (Analog Technologies, USA) with an applied voltage of 17 ± 0.5 kV and a needle‐to‐collector distance of 12 cm. The polymer solution was fed through the capillary by gravity, without the use of a syringe pump, ensuring a stable and continuous flow rate of approximately 1.0 mL/h. The electrospinning process was conducted for 30 min at 25°C ± 2°C and 30 ± 5% relative humidity. The fibers were collected on grounded aluminum foil and stored in a desiccator until characterization. The entire setup was enclosed to minimize air turbulence and ensure stable environmental conditions during fiber formation.

Samples were identified using acronyms to facilitate organization (Table 1). The acronym PCL refers to pure polycaprolactone. PCL‐E denotes samples containing red propolis extract and PCL, while PCL‐W refers to samples containing wax and PCL. The numerical suffix (e.g., 1, 5, 10, 15) indicates the percentage of extract or wax incorporated into the respective formulation.

**TABLE 1:** Sample abbreviations and proportions of each fraction PCL with red propolis extract and PCL with red propolis wax.

Samples	PCL (g)	Extract (g)	Wax (g)
PCL	1.8	—	—
PCL‐E1	1.782	0.018	—
PCL‐E5	1.71	0.09	—
PCL‐E10	1.62	0.18	—
PCL‐E15	1.53	0.27	—
PCL‐W1	1.782	—	0.018
PCL‐W5	1.71	—	0.09
PCL‐W10	1.62	—	0.18
PCL‐W15	1.53	—	0.27

### 2.3 Characterization Techniques

#### 2.3.1 Ultra‐Performance Liquid Chromatography

The isoflavonoid content of the red propolis extract and wax was determined using ultra‐performance liquid chromatography (UPLC) coupled with a diode array detector (UPLC‐DAD, Shimadzu, Japan). Separation was carried out using a C18 column (150 × 4.6 mm, 5‐µm particle size) [29].

#### 2.3.2 SEM and Porosity Analysis

The morphology of the electrospun fibers was analyzed using a VEGA3 scanning electron microscope (Tescan, Czech Republic) operating under high vacuum at a constant temperature, with an accelerating voltage of 20 kV and a secondary electron (SE) detector. Samples were mounted on stubs using carbon tape and sputter‐coated with a thin gold layer (Quorum Q150R ES) at 45 mA for 200 s. Fiber diameter distributions were evaluated using ImageJ software (NIH, USA). For each sample, 100 randomly selected fibers were measured to determine the average diameter, enabling quantitative morphological assessment.

#### 2.3.3 Wettability Analysis

The swelling behavior of the fiber mats was assessed by immersing circular samples (10 mm in diameter) in distilled water (pH 6.4), following the protocol described by Çay et al. [30]. Initial mass (*M*_initial_) was recorded, and samples were immersed in 50 mL of distilled water for time intervals ranging from 1 to 60 min. After immersion, excess surface water was carefully removed using absorbent paper, and the swollen mass (*M*_wet_) was recorded. Swelling tests were performed in triplicate. The degree of swelling was calculated using Equation (1) [30]:

(1) $$Degreeofswelling\left(\%\right)=\left(\left(M_{wet}\;-\;M_{initial}\right)/M_{initial}\right)\;\times \;100$$

Contact angle measurements were carried out using distilled water as the testing liquid. Samples were cut into 10 × 10 mm^2^ and placed on an adjustable alignment platform. A water droplet was deposited onto the sample surface using a 5 mL syringe equipped with a 25 × 0.6‐mm stainless steel needle. The measurement setup included a digital microscope (1600× magnification), an LED light source, and a connected computer. Images were captured 1–2 s after drop deposition, and contact angles were analyzed using ImageJ software.

#### 2.3.4 Cytotoxicity Assessment: Cytotoxicity Assessment

The murine fibroblast‐like cell line L929 (ATCC CCL‐1, Manassas, VA, USA) was purchased from ATCC. Cells were cultured on the surface of the biomaterials using Dulbecco's modified Eagle medium (DMEM, Gibco, Thermo Fisher Scientific, Waltham, MA, USA), supplemented with 100 IU/mL penicillin, 100 µg/mL streptomycin, 2 mmol/L glutamine (Gibco), and 10% fetal bovine serum (FBS; Gibco). After 24 h of incubation, cell viability was assessed using the Alamar Blue assay (Thermo Fisher Scientific). Before the assay, samples were washed with 1 mL of phosphate‐buffered saline (PBS) and subsequently incubated with 450 µL of DMEM (without FBS) and 50 µL of Alamar Blue solution for 4 h at 37°C in a 5% CO_2_ atmosphere. Two 100 µL aliquots of each condition were transferred to a 96‐well plate, and fluorescence was measured at 560 nm excitation and 590 nm emission wavelengths using a Synergy H1 microplate reader (BioTek, Winooski, VT, USA). The negative control (DMEM supplemented with 1% FBS) was considered 100% viable. Statistical analysis was performed, and differences were considered significant at *p* < 0.05 [31].

## 3 Results

### 3.1 Chromatographic Analysis

The chromatographic analysis identified peaks corresponding to flavonoids present in both the red propolis extract and wax samples. The retention times matched those of authenticated analytical standards and included liquiritigenin (12.48 min), daidzein (12.72 min), pinobanksin (16.04 min), isoliquiritigenin (17.20 min), formononetin (18.08 min), biochanin A (22.55 min), and pinocembrin (23.55 min). The analytical method proved effective for quantifying these compounds, with sensitivity in the range of 0.05–7.5 µg/mL.

The results demonstrated that liquiritigenin, daidzein, pinobanksin, isoliquiritigenin, formononetin, biochanin A, and pinocembrin were significantly more concentrated in the red propolis extract compared to the wax fraction, as summarized in Table 2. These findings are consistent with previous reports in the literature [21, 23, 29], which also characterized the phytochemical profile of red propolis extracts.

**TABLE 2:** Chromatographic analysis of flavonoid compounds in red propolis extract and wax samples.

Flavonoid	*λ*_max_ ^a^	Retention time (min)	Concentration (µg/mL)^b^	
			Extract	Wax
Liquiritigenin	275	12.48	5.050 ± 0.014	0.922 ± 0.001
2. Daidzein	249	12.72	2.574 ± 0.009	0.692 ± 0.004
3. Pinobanksin	289	16.04	0.955 ± 0.001	0.091 ± 0.009
4. Isoliquiritigenin	366	17.20	5.317 ± 0.011	1.658 ± 0.006
5. Formononetin	249	18.08	7.532 ± 0.105	4.070 ± 0.079
6. Biochanin A	249	22.55	0.578 ± 0.012	0.084 ± 0.002
7. Pinocembrin	291	23.55	0.606 ± 0.008	0.050 ± 0.001

^a^ Maximum absorption wavelength (nm).

^b^ Values represent mean ± standard deviation (*n* = 3). Retention times were consistent across replicate injections (variation < 0.1 min).

As anticipated, the wax fraction exhibited a markedly lower concentration of secondary metabolites. This difference is attributed to the purification process, during which thermal treatment may lead to partial degradation or loss of thermolabile bioactive compounds, thereby reducing their presence in the final wax product.

### 3.2 FTIR Analysis

The FTIR spectrum of the red propolis extract exhibited a broad absorption band in the 3600–3000 cm^−1^ range, with a maximum at approximately 3356 cm^−1^, attributed to the O─H stretching vibration of phenolic groups [23, 29, 32]. Additional low‐intensity bands were observed at 2971 and 2932 cm^−1^, corresponding to the symmetric and asymmetric stretching vibrations of CH_2_ and CH_3_ groups, respectively [20, 29, 33]. These bands may be associated with wax residues or terpenes present in the extract.

Characteristic peaks at 1619, 1507, and 1457 cm^−1^ were consistent with C═C stretching in the aromatic rings of flavonoids, corroborating the UPLC results. Phenolic compounds and flavonoids are also known to exhibit stretching and angular deformation bands in the narrow absorption range of 1300–1000 cm^−1^, including bands at 1157 cm^−1^ (*ν*C─O) and 1109 cm^−1^ (*δ*C─OH) [32]. The band at 1033 cm^−1^ corresponds to C─O stretching of aromatic ether groups, while the peak at 840 cm^−1^ is assigned to out‐of‐plane CH bending vibrations (γCH) [23, 34].

The FTIR‐ATR spectrum of the red propolis wax primarily revealed signals associated with aliphatic chains, consistent with its composition of esters, hydrocarbons, and fatty acids [35, 36]. Prominent absorption bands at 2916 cm^−1^ (*ν*asCH_2_) and 2846 cm^−1^ (*ν*sCH_2_) correspond to aliphatic hydrocarbon stretching. The spectral fingerprint region (1800–800 cm^−1^) showed characteristic lipid absorptions, including a band at 1733 cm^−1^ assigned to C═O stretching of cholesterol esters. Additional bands at 1464 cm^−1^ (*δ*asCH_2_), 1374 cm^−1^ (*δ*asCH_3_), and 1176 cm^−1^ (*ν*C═O and *δ*CH) were attributed to vibrational modes of fatty acids and esters. The weak band at 730 cm^−1^ is related to CH_2_ rocking vibrations typical of long‐chain hydrocarbons. The absence of bands associated with phenolic compounds, as detected in the extract, is likely due to their low concentration in the wax matrix.

**FIGURE 2:**
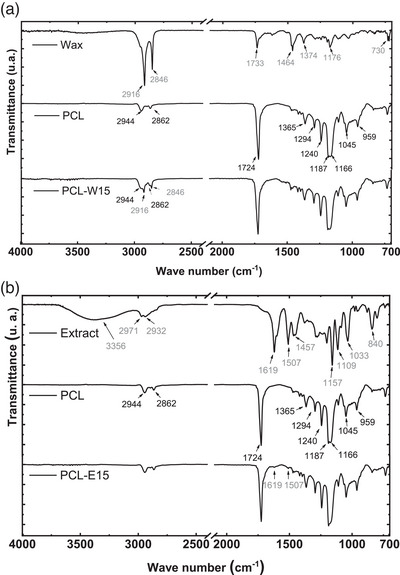
(a) FTIR spectra of red propolis extract, PCL, and PCL‐E15 mats. (b) FTIR spectra of red propolis wax, PCL, and PCL‐W15 mats.

The FTIR‐ATR spectra of PCL matrices containing 15% red propolis extract or wax retained the characteristic bands of pure PCL. Only mild contributions from propolis constituents were observed, suggesting either low additive concentration or possible overlap with PCL bands. Notably, no new bands emerged, indicating the absence of chemical bond formation between PCL and the added components, thereby confirming that the interaction was predominantly physical [37]. A similar outcome was reported by Azevedo et al. [26], who encapsulated red propolis extract in spherical forms and observed comparable spectral behavior (Figure 2).

### 3.3 SEM and Contact Angle

**FIGURE 3:**
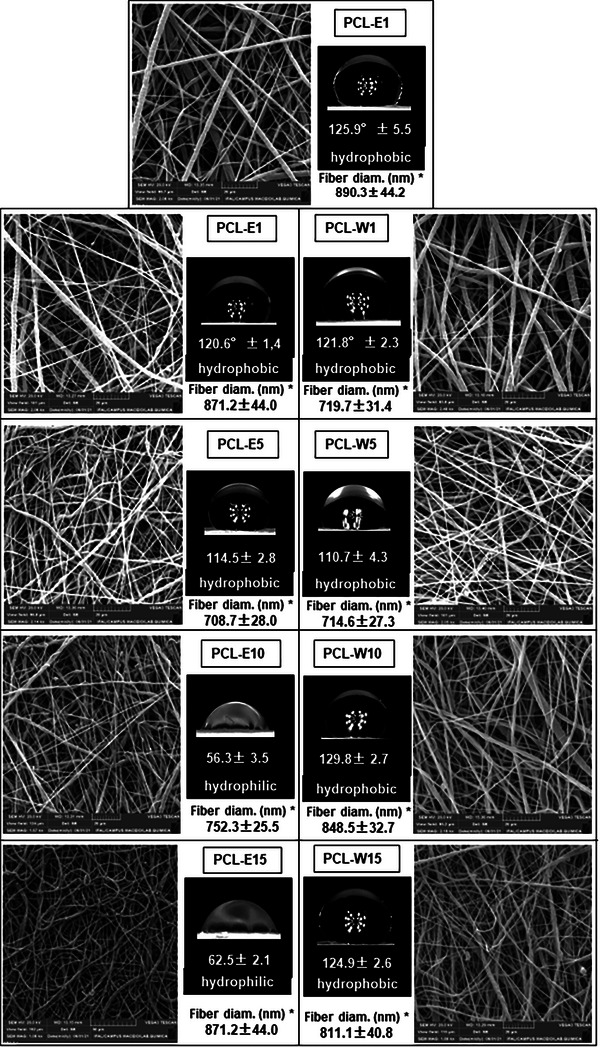
SEM micrographs and water contact angle measurements of electrospun PCL mats containing (top row) red propolis extract (PCL‐E) and (bottom row) red propolis wax (PCL‐W) at concentrations of 1, 5, 10, and 15 wt%. Each image is labeled with the respective formulation. Fiber diameters and contact angle values are expressed as mean ± standard deviation (*n* = 3). *Average fiber diameter.

The morphologies of the electrospun mats are shown in Figure 3, alongside their respective average fiber diameters and water contact angle measurements. SEM micrographs reveal that all samples exhibit continuous, predominantly smooth fibers, free of bead‐like defects, with a random distribution over the collector surface. The average fiber diameters ranged from 708.7 ± 28.0 nm for the sample containing 5% red propolis extract (PCL‐E5) to 890.3 ± 44.2 nm for the control sample (pure PCL) (*n* = 3), indicating that the incorporation of propolis fractions did not impair fiber formation, though slight thickening variations were observed.

The average fiber diameters for the samples containing red propolis extract were: PCL‐E1: 871.2 nm, PCL‐E5: 708.7 nm, PCL‐E10: 752.3 nm, PCL‐E15: 871.2 nm. For samples with propolis wax: PCL‐W1: 719.7 nm, PCL‐W5: 714.6 nm, PCL‐W10: 848.5 nm, PCL‐W15: 811.1 nm. All propolis‐containing samples (extract or wax) exhibited lower average diameters compared to the pure PCL mat. This reduction is attributed to a decrease in the viscosity of the electrospinning solutions, likely due to the lower molecular weight of the added fractions relative to PCL. This behavior aligns with the findings of Reshmi et al. [38], who reported that reduced viscosity leads to the formation of thinner fibers during electrospinning. Regarding wettability, contact angle measurements revealed distinct behaviors between formulations. The control PCL sample exhibited a contact angle of 125.9° ± 5.5°, confirming its hydrophobic nature. This characteristic was also maintained in the wax‐containing samples, with values of PCL‐W1: 121.8° ± 2.3°, PCL‐W5: 110.7° ± 4.3°, PCL‐W10: 129.8° ± 2.7°, PCL‐W15: 124.9° ± 2.6°. All values remained above 90°, classifying these materials as hydrophobic. In contrast, the extract‐containing samples exhibited a concentration‐dependent transition in surface wettability. PCL‐E1 remained hydrophobic (120.6° ± 1.4°), whereas PCL‐E10 (56.3° ± 3.5°) and PCL‐E15 (62.5° ± 2.1°) became markedly more hydrophilic. This shift is likely due to the migration of phenolic hydroxyl groups (─OH) from flavonoids to the fiber surface during electrospinning, increasing water affinity. This phenomenon is supported by the findings of Ravichandran et al. [39] and Viscusi et al. [13]. Collectively, the results presented in Figure 3 confirm that incorporating red propolis extract at concentrations ≥5% significantly alters the surface properties of electrospun PCL mats, promoting hydrophilicity. In contrast, the inclusion of red propolis wax preserves the inherent hydrophobicity of the PCL fibers.

The incorporation of the red propolis wax fraction, while maintaining the hydrophobic character of the PCL matrix, caused subtle morphological variations in the electrospun fibers. SEM micrographs showed a slightly less homogeneous fiber distribution in the wax‐containing samples, suggesting that the wax phase may partially segregate within the polymer jet during electrospinning, influencing local viscosity and charge density. This effect did not prevent fiber formation but may have contributed to small differences in surface roughness and fiber diameter variability.

From a functional perspective, the presence of the wax fraction tends to reduce water interaction, acting as a hydrophobic barrier that may slow down the diffusion of incorporated active compounds, suggesting a potential for slower release in wax‐containing formulations.

### 3.4 Swelling Degree

**FIGURE 4:**
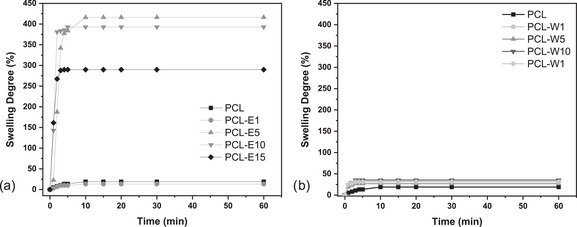
(a) Swelling profiles of electrospun PCL mats containing red propolis extract and (b) red propolis wax as a function of immersion time in distilled water.

Figure 4 presents the swelling profiles of electrospun mats as a function of immersion time in distilled water. The graph on the left corresponds to samples containing red propolis extract (PCL‐E), while the graph on the right displays the results for formulations with red propolis wax (PCL‐W). The data reveal distinct behaviors in water absorption capacity, reinforcing the findings obtained from the wettability analysis and FTIR spectra.

The samples PCL‐E5, PCL‐E10, and PCL‐E15 exhibited high swelling degrees, reaching saturation rapidly (within approximately 5–10 min), with values exceeding 350%, indicative of high hydrophilicity. This behavior is directly related to the reduced contact angles observed in Figure 3, with values below 90° for these same formulations, as well as to the presence of hydroxyl (─OH) functional groups identified in the FTIR spectra of the propolis extract (bands at ∼3356, 1157, and 1109 cm^−1^). These groups facilitate hydrogen bonding with water molecules, resulting in increased absorption. In addition, the PCL‐E5 sample, which presented the smallest average fiber diameter among the extract‐containing formulations (as shown in Figure 3), demonstrated the highest swelling degree. This may be attributed to the increased specific surface area, which enhances the exposure of hydrophilic groups to water, promoting diffusion through the fibrous matrix. Conversely, the samples containing red propolis wax (PCL‐W), regardless of concentration, maintained a low and consistent swelling degree (≤35%). This behavior is consistent with the hydrophobic nature evidenced by the contact angle measurements in Figure 3, where all PCL‐W formulations exhibited values above 120°. The FTIR spectra of the wax revealed the predominance of nonpolar aliphatic groups (CH_2_ and CH_3_), with strong absorption bands at 2916 and 2846 cm^−1^, and the absence of phenolic‐related bands, explaining the low surface affinity for water. These findings demonstrate that the presence of polar functional groups in the propolis extract plays a key role in modulating the wettability and swelling behavior of the mats, while the wax induces an apolar, hydrophobic, and poorly absorbent profile, even at higher concentrations.

Therefore, the data presented in Figure 4 corroborate the morphological and spectroscopic (FTIR) results, reinforcing that surface chemistry is a critical factor governing wettability and swelling behavior, with direct implications for biomedical applications. PCL‐E systems appear promising for absorbent wound dressings and tissue engineering, while PCL‐W systems are better suited for protective barriers or sustained drug release platforms.

### 3.5 Cytotoxicity

**FIGURE 5:**
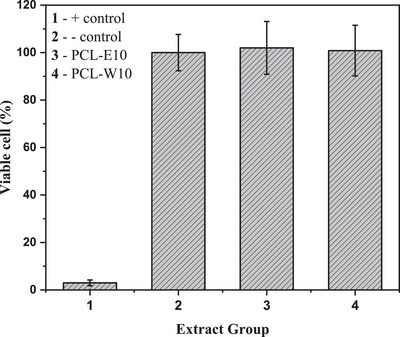
Cell viability of electrospun PCL, PCL‐E10, and PCL‐W10 mats evaluated using L929 fibroblast‐like cells and the Alamar Blue assay.

Cytotoxicity evaluation was conducted to determine the cell viability of the mats when exposed to living organisms. Cell viability was assessed based on the absorbance intensity generated by the reduction of the Alamar Blue reagent by metabolically active cells. Two mats were selected for testing: PCL‐E10 and PCL‐W10, which contain 10% of red propolis extract and wax, respectively. The results of the cytotoxicity assessment are illustrated in Figure 5.

The positive control exhibited less than 10% cell viability, as expected, due to the absence of viable cells. Examination of the graph (Figure 5) revealed 100% cell viability for both PCL‐E10 and PCL‐W10 samples. According to the ISO 10993‐5:2009 standard, this result classifies the electrospun mats as noncytotoxic, meaning they exhibit no cellular reactivity. Cell cultures presented only discrete cytoplasmic granulation, with no evidence of cell lysis or growth inhibition. In other words, the materials are biologically inactive and safe for use in contact with living tissues.

The results also suggest that the developed systems are thermally and structurally stable under conditions typically required for biomedical use. This inference is supported by recent evidence from Thomé et al. [40], who demonstrated that gamma irradiation at 29.91 kGy effectively sterilizes electrospun PCL mats containing Brazilian red propolis without compromising morphology, chemical integrity, or antioxidant activity, while enhancing polymer crystallinity due to molecular chain reorganization. Such stability under sterilization and storage conditions reinforces the suitability of these materials for clinical and pharmaceutical applications.

## 4 Conclusion

This study comprehensively demonstrated that the incorporation of different red propolis fractions—extract and wax—into electrospun PCL mats enables selective modulation of morphological, chemical, and functional properties without compromising the system's biocompatibility. Chromatographic analysis (UPLC–DAD) revealed that red propolis extract contains significantly higher concentrations of bioactive flavonoids, such as formononetin, liquiritigenin, and isoliquiritigenin, compared to the wax fraction, which exhibits a predominantly aliphatic composition. This compositional difference was confirmed by FTIR spectra, which showed phenolic hydroxyl groups in the extract‐based formulations, whereas the wax‐based samples displayed characteristic bands of apolar chains such as CH_2_ and esters. SEM images demonstrated that the electrospinning process yielded continuous, uniform, bead‐free fibers with average diameters below 900 nm across all samples. The addition of extract induced slight alterations in surface roughness and, in some formulations, a reduction in fiber diameter—an effect attributed to increased specific surface area. In contrast, wax incorporation resulted in a slight thickening of the fibers, possibly due to its higher viscosity and resinous nature. Contact angle measurements revealed a marked hydrophilic transition in mats containing ≥5% extract, with values falling below 90°, whereas all wax‐containing formulations remained hydrophobic (angles >120°). This trend was reflected in the swelling results, where extract‐based mats absorbed up to 400% water, indicating high affinity with aqueous environments—an effect linked to the presence of polar functional groups in flavonoids. Conversely, wax‐containing mats exhibited low water absorption capacity, consistent with their predominantly lipophilic composition. Finally, cytotoxicity assays confirmed that both extract‐ and wax‐containing mats were noncytotoxic, demonstrating biological safety for biomedical contact.

The developed materials exhibited physicochemical robustness consistent with stable performance under sterilization and storage conditions, supporting their potential use in biomedical applications. In summary, the incorporation of red propolis extract enables the development of hydrophilic, absorbent, and bioactive biomaterials suitable for smart dressings and regenerative scaffolds, while the addition of wax provides hydrophobic, moisture‐resistant surfaces with potential for protective barriers or sustained drug release. These results demonstrate that tailoring the type of propolis fraction is an effective and customizable strategy to adapt biomaterial functionality to specific clinical applications.

## Funding

CNPq (Grant numbers 429027/2018‐4 and 305893/2022‐0) and FAPEAL (Grant number E:60030.0000001210/2024).

## Conflicts of Interest

The authors declare no conflicts of interest.

## Data Availability

The authors have nothing to report
